# Comorbidities and Treatment Outcomes in Patients Diagnosed with Drug-Resistant Tuberculosis in Rural Eastern Cape Province, South Africa

**DOI:** 10.3390/diseases12110296

**Published:** 2024-11-19

**Authors:** Mojisola Clara Hosu, Lindiwe Modest Faye, Teke Apalata

**Affiliations:** 1 Department of Laboratory Medicine and Pathology, Faculty of Medicine and Health Sciences, Walter Sisulu University, Private Bag, Mthatha 5117, South Africa; lfaye@wsu.ac.za (L.M.F.); tapalata@wsu.ac.za (T.A.); 2 National Health Laboratory Service, Mthatha 5099, South Africa

**Keywords:** drug-resistant tuberculosis, treatment outcomes, comorbidity, hearing loss, hypertension, non-communicable diseases, logistic regression

## Abstract

Background/Objectives: Tuberculosis (TB) remains a significant global health challenge, with drug-resistant tuberculosis (DR-TB) posing a greater threat due to difficulty in treatment. This study aimed to investigate the relationship between comorbidities and treatment outcomes in patients diagnosed with DR-TB in rural Eastern Cape using logistic regression. Methods: Data on patient characteristics, comorbidities, and treatment outcomes were extracted from the medical records and analyzed using Python version 3.8. and R version 4.1.1 software. A logistic regression model was used to determine the effects of selected variables on treatment outcomes of DR-TB cases. Results: Hearing loss and hypertension (HTN) were the most frequently observed comorbidities across various DR-TB cases, particularly rifampicin-resistant (RR), multidrug-resistant (MDR), and pre-extensively drug-resistant (pre-XDR-TB) cases. A hearing loss prevalence of 5.8% (26/445) was found among patients receiving treatment for TB, with the intensity of impairment ranging from mild to severe. Gender is significantly associated with the occurrence of HTN among these patients (*p*-value: 0.022). Comorbidities such as epilepsy, hearing loss, and HTN significantly impact treatment success, with higher risks of mortality and incomplete cure. Using logistic regression, obesity (OR = 3.0884; e = 1.1277; *p* = 0.0408) and HIV-positive status (OR = 0.4458; e = 0.8078; *p* = 0.0001) were highly likely and less likely associated with better treatment outcomes, respectively. The logistic regression model achieved an accuracy of 64.0%, a precision of 63.0%, and a recall of 95.0%, with an F-1 score of 76.0%. Conclusions: The findings underscore the importance of implementing integrated management strategies that address both DR-TB and its comorbidities, particularly in resource-limited settings where such patients are prevalent. Public health policies should incorporate strategies to provide nutritional assessments and interventions, particularly for individuals with low BMI. This could include food supplementation programs or partnerships with local food kitchens to ensure that patients have access to adequate nutrition during DR-TB treatment.

## 1. Introduction

Non-communicable diseases (NCDs), responsible for two-thirds of fatalities globally, of which 77% are in low- and middle-income countries (LMICs) [1,2], represent an urgent and growing global public health emergency in terms of both the human suffering they cause and the havoc they wreak on the socioeconomic fabric of countries [3]. This situation in Sub-Saharan Africa (SSA) is concerning, particularly with the strain of the heavy burden of endemic diseases such as tuberculosis (TB) and human immunodeficiency virus (HIV) [4,5,6]. The most common chronic NCDs reported globally include cardiovascular diseases, diabetes, cancer, and chronic respiratory diseases [1,7]. Cardiovascular diseases account for most NCD deaths (17.9 million) annually, followed by cancers (9.3 million), chronic respiratory diseases (4.1 million), and diabetes (2.0 million, including kidney disease deaths caused by diabetes) [3]. The rapid increase in NCDs is driven by a combination of factors, including an ageing population, rapid urbanization, fluctuations in environmental factors, and lifestyle modifications [8,9]. As populations live longer due to improved living standards and healthcare, the proportion of elderly individuals increases. Older age is a major risk factor for many NCDs, such as cancer, cardiovascular diseases, and dementia. Comorbid NCDs with TB commonly reported include hypertension, diabetes mellitus, cancer, and chronic obstructive pulmonary diseases (COPD) [10]. NCDs threaten progress towards the 2030 Agenda for Sustainable Development Goals, which includes a target of reducing premature deaths from NCDs by one-third by 2030 [11]. One of the strategic pillars of the EndTB strategy incorporates integrated, patient-centered TB care and prevention, the key component of which focuses on collaborative TB/ HIV activities and the management of comorbidities [12].

South Africa faces a quadruple burden of disease resulting from maternal and child mortality: communicable diseases such as HIV/AIDS and tuberculosis (TB); NCDs such as hypertension and cardiovascular diseases, diabetes, cancer, mental illnesses, and chronic lung diseases such as asthma; as well as injury and trauma. Significant socioeconomic inequities in South Africa result in a higher chronic disease burden and mortality among poorer people [1,13]. A study in a primary healthcare facility in the Western Cape reported the rates of NCD comorbidity to be as high as 65% [14]. Another study with a report from primary healthcare facilities in four provinces in South Africa found that the combination of diabetes and hypertension was the commonest comorbid condition [15].

The results of various studies have indicated that different comorbidities, including HIV infection, significantly impact TB treatment outcomes, treatment success rates, and mortality associated with tuberculosis [16]. The findings of various studies indicated that host factors, including smoking, alcohol abuse, low body mass index (BMI), comorbidity such as HIV infection, diabetes mellitus (DM), chronic renal failure (CRF), malignancy, and chronic obstructive pulmonary disease (COPD), are risk factors for the development of TB [17,18]. Understanding risk factors for DR-TB mortality is critical to improving DR-TB treatment outcomes. Previous studies have reported that cigarette smoking, comorbidities including HIV/AIDS and diabetes, and therapeutic delay contribute to DR-TB mortality [19,20,21]. Several of these factors have been significantly associated with poor treatment outcomes [22,23], and the development of MDR-TB [18,24]. Comorbidities are critical factors in the control of TB. Hence, improving the health status of the patient with the timely detection, management, and effective treatment of comorbidities may reduce the development of TB and the spread of DR-TB and promote good treatment outcomes.

Therefore, this study was conducted to investigate the relationship between treatment outcomes in patients diagnosed with drug-resistant tuberculosis and concomitant comorbidities in the rural Eastern Cape Province of South Africa.

## 2. Materials and Methods

### 2.1. Study Design and Population

This retrospective cohort study accessed patients’ medical records diagnosed with DR-TB in a rural municipality of the Eastern Cape. The study population consisted of patients notified between 2018 and 2020. Patients with missing or incomplete data on comorbidities or treatment outcomes were excluded from the analysis. Finally, after the data curation process described above, patients who met the criteria of having confirmed DR-TB, documented comorbidity, and a recorded treatment outcome were included in the analysis.

### 2.2. Data Collection

Data on patient characteristics, comorbidities, and treatment outcomes were extracted from the medical records. The presence of comorbidities was assessed based on the International Classification of Diseases; Eleventh Revision (ICD-11) codes recorded in the registry. These comorbidities were considered the primary comorbidities by the attending physicians. These categories were also used for further analysis. BMI, including underweight (<18.5 kg/m^2^), normal weight (18.5–25 kg/m^2^), overweight (≥25 kg/m^2^), and obese (≥30 kg/m^2^), was used in our analysis.

### 2.3. Outcome Categories

Treatment outcomes were mainly categorized according to the WHO definitions, including treatment success (cured or treatment completed), treatment failure, death, lost to follow-up (LTFU), and not evaluated (transferred out) [25]. In clinical practice and surveillance, these standardized categorizations allow for monitoring the effectiveness of TB treatment, giving individualized care to the patient, and measuring the effectiveness of public health strategies.

According to the 2021 updated WHO treatment outcome [25] definitions for TB, the following categories were used:

Cured: A pulmonary TB patient is declared cured when treatment is completed with an indication of bacteriological response and no evidence of failure.

Treatment completed: A patient who completed treatment as recommended but whose outcome does not meet the definition of cure or treatment failure.

Treatment failure: A patient whose treatment routine got terminated or permanently switched to a new treatment strategy due to poor clinical response, adverse drug reaction, or evidence of additional drug resistance.

Died: A patient who is demised before treatment is initiated or during treatment.

LTFU: A patient who did not commence treatment or whose treatment was interrupted for two consecutive months or more.

Not evaluated: A patient for whom no treatment outcome was allotted, including transferred out and excluding LTFU patients.

### 2.4. Statistical Analysis

Data collected were coded, entered, and analysed using Python version 3.8. and R version 4.1.1 software. A *p* < 0.05 was considered to be significant. Categorical data were presented as frequencies and percentages, while continuous data were presented in the form of mean and SD. The independent variables were age, gender, residence, HIV status DR-TB type, comorbidities, and BMI. The main outcome variable, DR-TB treatment outcome was categorised into successful (cured and completed) and unsuccessful (treatment failure, died, and LTFU) treatment outcomes, and scored as follows: successful treatment = 1, unsuccessful treatment = 0. The secondary outcome variable, the presence of comorbidities, was indicated based on the clinician’s assessment and evaluation. Bivariate analysis was used to determine any significant association between independent variables and the treatment outcome. The variables showing significant association were used in logistic regression. The Generalized Linear Regression model (GLM) was used to determine the effects of selected variables on the treatment outcomes of DR-TB cases. These variables included BMI (underweight, normal weight, overweight, and obesity) and HIV status. The GLM model is a class of linear-based regression models used to model binary or count data. Specifically, a logistic regression model was used since the outcome (successful vs. unsuccessful treatment) is binary. The model allowed us to build a linear relationship between the response and predictors, even though their underlying relationship was not linear. This is possible using a link function that connects the response variable to a linear model. This is shown in the formula below:

$$\log\;\left(\;\frac{P}{1-P}\right)\;=\;\beta_{0}\;+\;\beta_{1}X_{1}\;+\;\beta_{2}X_{2}\;+\;\ldots \;+\;\beta_{n}X_{n}$$

where *P* is the probability of the event occurring (e.g., the probability of a successful treatment outcome),

log$\left.\left(\;\frac{P}{1-P}\right.\right)$ is the log-odds or logit of the probability,

β_0_ is the intercept

β_1_, β_2_, …, β_n_ are the coefficients for the independent variables X_1_, X_2_, …, X_n_, which are linked linearly to the log-odds.

A multivariate logistic regression analysis was carried out with treatment outcomes being the dependent variable, while the independent variables were BMI categories (underweight, normal weight, overweight and obesity), and HIV status.

## 3. Results

### 3.1. Characteristics of the Study Participants and Comorbidities

A total of 445 patients were enrolled in the study, of which 56% were males and 44% females. The study population’s mean age (±SD) was 37.7 (±12.7) years. Females are notably underrepresented among DR-TB patients, implying that females have a lower risk or are less likely to be diagnosed with DR-TB in this region.

### 3.2. Distribution of DR-TB

The distribution of DR-TB cases in Table 1 below indicates that RR-TB accounts for the majority with 46.1% of cases, closely followed by MDR-TB at 43.6%. Less prevalent forms are Pre-XDR-TB at 5.2%, XDR-TB at 3.8%, and INHR-TB at 1.3%.

### 3.3. Comorbidities Stratified According to DR-TB Type

**Figure 1:**
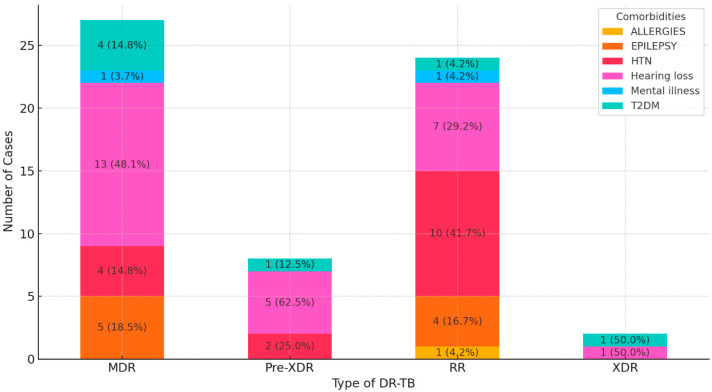
Distribution of comorbidities in DR-TB patients.

A total of six comorbidities were associated with DR-TB in the study’s population, including diabetes mellitus type 2 (T2DM), hypertension (HTN), hearing loss, epilepsy, mental illness, and allergies (Figure 1). Hearing loss and HTN are the most frequently observed comorbidities across the various types of DR-TB, particularly in MDR, RR, and Pre-XDR cases. Epilepsy and T2DM are also observed but to a lesser extent. Comorbidities such as mental illness and allergies are less common across all types of DR-TB. This distribution suggests that managing comorbidities such as hearing loss and HTN is critical for patients with DR-TB, especially for MDR and RR forms.

### 3.4. Correlation of Comorbidities with Demographics

#### 3.4.1. Age

HTN and allergies are most prevalent in older individuals, with average ages of 56.5 and 56 years, respectively. Mental illness and hearing loss are more common in younger individuals, with average ages of 32 and 36.3 years.

#### 3.4.2. Education

T2DM is most common among individuals with no formal education (57%), while hearing loss and HTN show higher prevalence among individuals with primary and secondary education. Epilepsy and mental illness are more evenly distributed across education levels.

#### 3.4.3. Income Source

HTN and T2DM are more frequent among individuals with no income or dependent on government grants. Hearing loss and epilepsy are more common in individuals earning wages or casual labour.

#### 3.4.4. Occupation

Allergies are predominantly seen in individuals working in the private sector (100%).

HTN and T2DM are also observed in individuals with varied occupations, with a notable presence in government departments and pensioners.

Conclusively, age and gender show notable differences across comorbidities. For example, HTN and T2DM affect older females, while epilepsy and hearing loss are more prevalent among younger males. Education and income levels appear to influence the presence of certain comorbidities, especially with HTN and T2DM among individuals with no income or formal education. The *p*-values obtained when comparing gender groups (male vs. female) across different comorbidities are as follows: allergies (*p* = 1.0) show no significant difference, epilepsy (*p* = 0.326) is not significant, HTN (*p* = 0.022) shows a significant difference between genders, hearing loss: (*p* = 0.237) is not significant, mental illness (*p* = 1.0) shows no significant difference, and T2DM (*p* = 0.741) is not significant. The only comorbidity with a statistically significant gender difference is HTN, indicating that gender is likely to influence the occurrence of HTN among these patients.

### 3.5. Impact of Comorbidities on Treatment Outcomes

The impact of comorbidities on treatment outcomes was analyzed by categorizing outcomes as either successful or unsuccessful, using the following definition:

Successful outcomes include patients who were cured or completed treatment.

Unsuccessful outcomes include patients who died, had treatment failure, were LTFU, or transferred out.

#### 3.5.1. Cured Rates

Over 55% of patients with epilepsy were cured, indicating a relatively positive treatment outcome. Approximately 38% of patients with hearing loss were cured. Among patients with HTN, 26.6% achieved a cure, showing a moderate cure rate compared to other comorbidities. While with mental illness, 50% were cured, suggesting that mental illness did not significantly hinder treatment success.

#### 3.5.2. Treatment Completion

Patients with allergies had a 100% treatment completion rate, indicating strong adherence to or effective treatment for this group. A significant 53% of patients with HTN completed treatment without being fully cured. This suggests that many patients reached the end of treatment but may not have achieved complete recovery. For patients with hearing loss, 19% of patients completed treatment, with a mix of both cured and non-cured outcomes.

#### 3.5.3. Mortality

Epilepsy and HTN both show higher mortality rates, with 22% and 13% of patients dying during treatment, respectively. In patients with hearing loss, 11.5% of the patients died, indicating that this comorbidity is associated with higher mortality risk.

#### 3.5.4. Other Negative Outcomes

**Figure 2:**
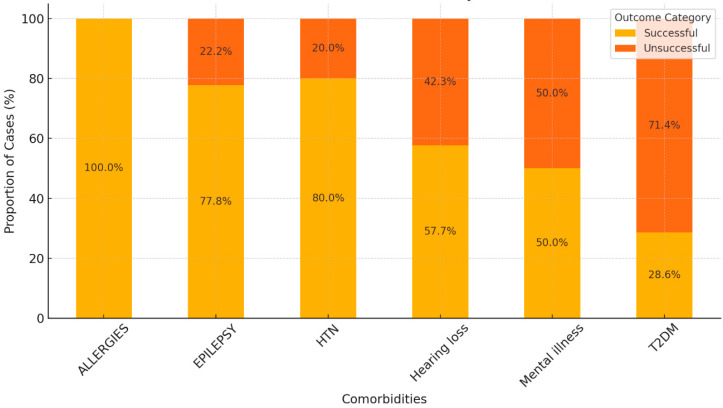
Treatment outcomes stratified by comorbidities.

Hearing loss and HTN patients were LTFU or experienced treatment failure at moderate rates. For those with “transferred out” outcomes, 50% of patients with mental illness were transferred out, indicating possible relocation or the continuation of treatment elsewhere, but not necessarily due to treatment failure. For epilepsy and hearing loss, while both conditions show significant cure rates, they are also associated with elevated mortality rates. This suggests that patients with these conditions are at higher risk of death, even when treatment is initiated. For HTN, although many patients with hypertension completed treatment, the relatively high mortality and incomplete cure rates suggest that hypertension could complicate TB treatment outcomes. Those with mental illness showed a mixed result, with high cure rates but with a large proportion of patients being transferred out, likely due to continued need for care. Patients with allergies showed the best outcome, with all patients completing treatment (Figure 2).

Overall, comorbidities such as epilepsy, hearing loss, and HTN significantly impact treatment success, with higher risks of mortality and incomplete cure. Managing these conditions alongside TB treatment is crucial for improving overall patient outcomes.

### 3.6. Impact of Comorbidities on Treatment Outcomes Stratified by HIV Status

#### 3.6.1. Allergies

**Figure 3:**
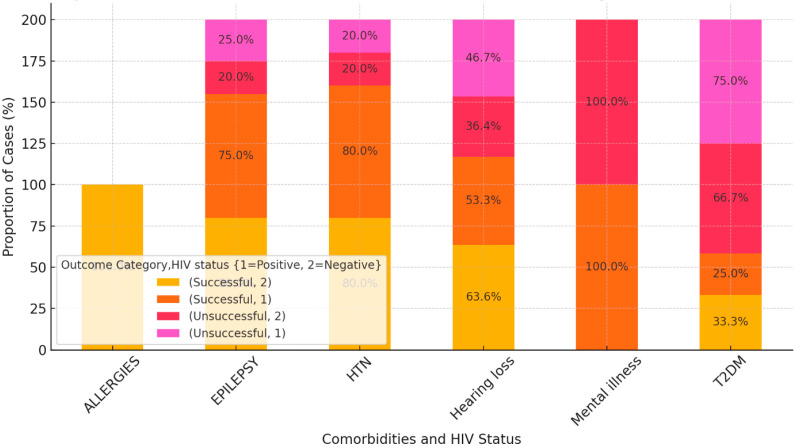
Impact of comorbidities on treatment outcomes stratified by HIV status.

HIV-negative patients with allergies had a 100% success rate in terms of treatment, with no unsuccessful outcomes observed (Figure 3).

#### 3.6.2. Epilepsy

HIV-negative patients had an 80% success rate, while 20% experienced unsuccessful outcomes. HIV-positive patients had a slightly lower success rate at 75%, with 25% experiencing unsuccessful outcomes. There was a slight difference in treatment success between HIV-positive and negative patients, but the impact of epilepsy remained significant for both groups.

#### 3.6.3. HTN

Both HIV-negative and HIV-positive patients with hypertension had the same outcomes, with 80% achieving successful treatment and 20% facing unsuccessful outcomes. HIV status does not appear to influence treatment outcomes significantly for hypertensive patients.

#### 3.6.4. Hearing Loss

The majority of HIV-negative patients had successful outcomes (57.7%), while 42.3% had unsuccessful treatment outcomes. In HIV-positive patients, hearing loss also presented significant risks, but no clear data were available in this analysis.

#### 3.6.5. Mental Illness

HIV-negative patients with mental illness had an equal chance of success and failure (50% successful and 50% unsuccessful outcomes).

Patients with allergies and HTN comorbidities tended to have better treatment outcomes, regardless of HIV status. Comorbidities of epilepsy and hearing loss posed a greater risk, with significant proportions of both HIV-positive and negative patients experiencing unsuccessful outcomes. DR-TB patients with mental illness were associated with poor outcomes, particularly for HIV-negative patients, where half of the patients experienced unsuccessful treatment outcomes.

Overall, HIV status did not seem to have a major impact on treatment outcomes across most comorbidities, with similar success and failure rates observed for both HIV-positive and negative patients. However, the comorbidity type plays a crucial role in determining the likelihood of successful treatment.

### 3.7. Comparison of Treatment Outcomes Across DR-TB Types

**Figure 4:**
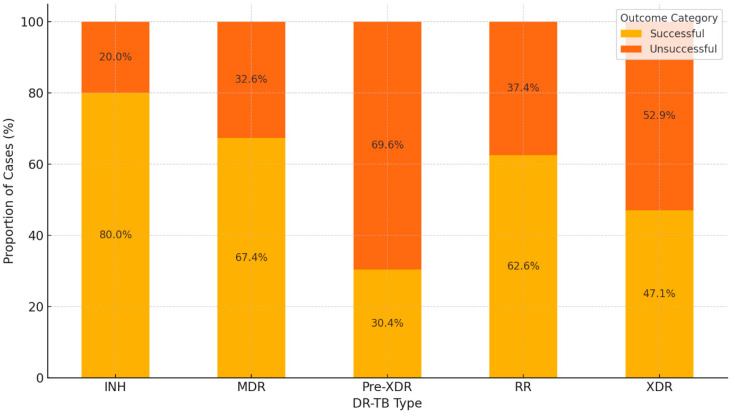
Comparison of treatment outcomes across DR-TB types.

Figure 4 shows the comparison of treatment outcomes (successful vs. unsuccessful) across different DR-TB types. In RR-TB and MDR-TB, there is a relatively balanced distribution of successful and unsuccessful outcomes, with a notable portion of successful treatments. In pre-XDR TB, there is a higher proportion of unsuccessful outcomes compared to MDR and RR, indicating more treatment challenges. In the XDR group, a larger proportion of unsuccessful outcomes were observed, reflecting the severity of extensively drug-resistant TB. This result indicates that as TB resistance increases (from MDR to XDR), the likelihood of successful outcomes decreases.

**Figure 5:**
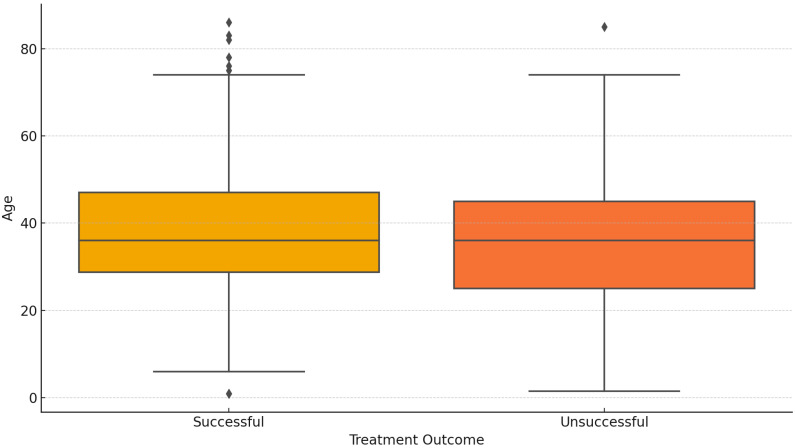
Age distribution across treatment outcomes.

None of the DR-TB types show a statistically significant difference in outcomes based on age, as all *p*-values are greater than the typical significance threshold of *p* ≤ 0.05 (Figure 5).

**Figure 6:**
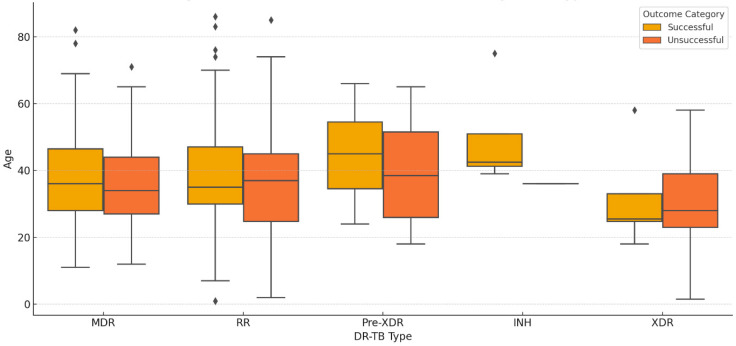
Age distribution across treatment outcomes by DR-TB type.

None of the DR-TB types show significant differences in age between successful and unsuccessful outcomes, as all *p*-values are greater than the typical threshold of *p* < 0.05 (Figure 6).

Age appears to have a limited impact on treatment outcomes across most DR-TB types, as the differences in average age between successful and unsuccessful outcomes are small, and the *p*-values indicate no statistically significant effects. However, older patients in the pre-XDR and INH groups might have slightly better outcomes compared to younger patients, though these effects are not conclusive.

### 3.8. Logistic Regression Model on Treatment Outcomes

DR-TB types and outcomes (target variables) include successful (1) versus unsuccessful (0). Table 2 shows the result of the logistic regression model.

For those in the BMI overweight category, the coefficient is −0.1481 and OR = 0.8623. The coefficient of −0.1481 indicates a negative association between being in the overweight BMI category and having a successful treatment outcome. This means that individuals who are overweight have 14% lower odds of a successful treatment outcome compared to those with a normal weight, and the association is not statistically significant (*p* = 0.4). For patients who are in the obese category, a coefficient of 1.1277 indicates a positive association between being categorized as obese and the likelihood of having a successful treatment outcome for DR-TB. The odds ratio of 3.0884 means that individuals categorized as obese are approximately three times more likely to experience a successful treatment outcome compared to those with normal weight, and the association is statistically significant (*p* = 0.04). The underweight category with a coefficient of −0.2964 indicates a negative association between being categorized as underweight and the likelihood of having a successful treatment outcome for DR-TB. An odds ratio of 0.7435 means that individuals categorized as underweight have approximately 25.7% lower odds of experiencing a successful treatment outcome compared to those who have normal weight. A *p*-value of 0.0394 indicates a statistically significant association. For HIV-positive patients with a coefficient of −0.8078 and OR = 0.4458, it means that HIV-positive patients have approximately 55.4% lower odds of successful treatment compared to HIV-negative patients.

The model performed with an accuracy of 63.0%, a precision of 70.0%, a recall of 95.0%, and an F1-score of 76.0%.

BMI and HIV status are the most significant factors influencing treatment outcomes. Being obese improves the likelihood of success, while HIV-positive patients are less likely to have better outcomes.

## 4. Discussion

The presence of comorbidity differs markedly from underlying infections. However, it can impact medication administration, patient management, and TB treatment outcomes [26]. Severe manifestations of comorbidities can determine the prognosis of the underlying infection and its subsequent course [27]. The findings of this study contribute to the growing body of evidence on the impact of comorbidities on TB treatment outcomes and mortality rates. Our results indicate that hearing loss and HTN were the most frequently observed comorbidities across various DR-TB, particularly in RR-TB, MDR-TB, and pre-XDR-TB cases. A study by Starshinova et al. [26] assessed comorbidities among patients with MDR-TB and XDR-TB, highlighting that chronic conditions such as HTN and cardiovascular diseases significantly impacted treatment outcomes. A retrospective study conducted in Shandong, China, reported a 16.3% prevalence of comorbidity among retreated PTB cases of the DR-TB [28], similar to our study, with a 13.7% prevalence of comorbidities.

The highest proportion of comorbidity was found for DM (9.5%), followed by HTN (2.0%) and COPD (1.8%), among patients with re-treated PTB by Tao et al. [28]. The comorbidities were categorized into pulmonary and extra-pulmonary comorbidities. Hearing loss (5.8%) was the most prevalent comorbidity, followed by HTN (3.6%), epilepsy (2.0%), and T2DM (1.6%). While extrapulmonary comorbidities such as HIV infection, chronic kidney disease (CKD), and DM weaken the immune system as they facilitate the development of TB [29,30], on the other hand, pulmonary comorbidities such as COPD promote the development of TB by destroying inherent lung defense, impairing lung function, and changing the form of the lung [31,32]. DM, a risk factor for TB, accounts for 6–24% of the TB burden depending on geographical differences [33]. The effect of DM on active TB is such that it interferes with the absorption and clearance of medications and increases the bacillary burden in patients with active TB, thereby extending the culture conversion duration and treatment.

Hearing loss (HL) is the fourth-highest cause of disability globally, with an estimated annual cost of over 750 billion dollars, resulting in social isolation, loneliness, stigma, and loss of productivity at the individual level, putting employment stability and family prosperity at risk and impacting society and the economy [34,35,36,37]. Disabling hearing loss (DHL) refers to hearing loss greater than 35 decibels (dB) in the better-hearing ear. Nearly 80% of people with disabling hearing loss live in low- and middle-income countries [37]. In the absence of intervention, the World Health Organization (WHO) projects that by 2030 there will be close to 630 million individuals suffering from DHL, and by 2050 that figure may reach over 900 million [34,36,37]. Prior to 2018, second-line injectable drugs (SLIDs), including amikacin, kanamycin, and capreomycin, were part of the guidelines recommended for MDR-TB treatment by the WHO. These drugs cause significant adverse events, especially nephrotoxicity and ototoxicity, with ototoxicity being irreversible [34,38,39,40]. HL-induced ototoxicity is a risk factor contributing to the current prevalence of HL worldwide. For the treatment of DR-TB, statistics indicate ototoxicity from aminoglycosides, which directly causes HL rates ranging from 10 to 50% [41]. A recent systematic review conducted by Dillard et al. [40] reported an estimated ototoxic HL prevalence of 41% in DR-TB treatment. According to Hong et al. [42], HL is the most common reason for suspending aminoglycoside (AG) treatment, which raises the risk of treatment breakdown and additional DR-TB transmission in the home and community. AG-induced HL aggregate incidence varies between 24% and 69% for DR-TB-infected individuals in South Africa [43]. In our study, although HL was present as a comorbidity in 5.5% of our participants, AG-induced HL was detected in 38% of our participants, while a study by Harris et al. [39] conducted in the Western Cape of South Africa detected HL in 57% of patients. AG-induced HL has been reported in MDR-TB patients during the injectable treatment phase, which may occur with or without tinnitus due to permanent damage to the outer hair cells in the cochlea and can progress even with cessation of AG treatment [35,44]. Medications such as kanamycin, a SLID for treating patients with DR-TB, have been reported to suppress cochlea activity, leading to ototoxicity and irreversible bilateral HL [44,45]. Ototoxicity refers to the functional impairment of the inner hair cells and nerves caused by specific medications and chemical substances [46]. AG ototoxicity leads to poor treatment outcomes [46,47]. Hence, the WHO has proposed the replacement of AGs with oral substitutes such as bedaquiline. The findings of Khoza-Shangase and Prodromos [44], Khoza-Shangase [48], and Modongo et al. [49] agree with our findings of HL in DR-TB patients, including the risk of ototoxicity increasing with increased duration of AG therapy. Managing comorbidities such as HL is critical for patients with DR-TB, particularly in RR-TB and MDR-TB cases. An age- and gender-matched study by Khoza-Shangase and Prodromos [44] demonstrated the positive benefits of bedaquiline over AG in that it did not lead to ototoxicity in the patients who were administered the drug. The implementation of an appropriate ototoxicity surveillance plan is vital to reduce patients’ chances of experiencing HL. The risk of the onset and progression of HL can be decreased by making sure clinicians are aware of the ototoxic effects of these drugs and by implementing audiological evaluation for the early detection of HL [3]. Furthermore, Khoza-Shangase [50] recommended that an ototoxicity monitoring protocol should be implemented within this population to collate systematic data for the adoption of evidence-based protocols within the South African context for the early detection and intervention of ototoxic HL. An analysis of the current trends in HL underscores the significance of implementing explicit policy recommendations and increasing public awareness of the condition to prevent and reduce its effects in the future [34].

Epilepsy and HTN both show higher mortality rates, with 22% and 13% of patients dying during treatment, respectively, while with HL, 11.5% of patients died, indicating that this comorbidity is associated with higher mortality risk.

Using the logistic regression model, BMI and HIV status were the most significant factors influencing treatment outcomes. A higher BMI improves the likelihood of success, while HIV-positive patients are less likely to have better outcomes. The model performed with an accuracy of 63.0%, a precision of 70.0%, a recall of 95.0%, and an F-1 score of 76.0%. The association between higher BMI and improved treatment outcomes suggests that nutritional support and intervention should be a priority in TB and HIV treatment programs. Healthcare providers should receive training to recognize the importance of BMI in DR-TB treatment outcomes, thereby enhancing patient management. This includes educating providers on the need for nutritional counselling and monitoring BMI as part of routine care for TB/HIV co-infected patients. Public health policies should incorporate strategies to provide nutritional assessments and interventions, particularly for individuals with low BMI. This could include food supplementation programs or partnerships with local food kitchens to ensure that patients have access to adequate nutrition during treatment. The association between being HIV-positive and unsuccessful treatment outcomes should guide healthcare policymakers in resource allocation. Healthcare facilities may need to focus more on supporting HIV-positive patients with additional resources, such as improved counseling services and access to ART, enhanced monitoring, and adherence support to mitigate their risk of unsuccessful treatment outcomes.

## 5. Conclusions

Our results indicate that the presence of comorbidities such as HL, HTN, and epilepsy is associated with unsuccessful treatment outcomes. However, the regression analysis indicated that being obese was significantly associated with successful treatment outcomes, and being HIV-positive was significantly associated with unsuccessful treatment outcomes among patients diagnosed with DR-TB in rural Eastern Cape. Our study underscores the significance of addressing comorbidities in TB management. Public health campaigns in the Eastern Cape should focus on raising awareness about the risks associated with low BMI and untreated HIV in the context of TB treatment. Educating local communities about the importance of nutrition and regular health check-ups should be prioritized, as it will empower individuals to seek care proactively. These findings emphasize the importance of personalized care strategies that consider the unique challenges faced by different patient populations. By recognizing obesity as a potential positive prognostic factor and addressing the complexities associated with HIV co-infection, healthcare providers can enhance treatment effectiveness and improve overall patient outcomes in managing DR-TB. Integrated TB and comorbidity care must be prioritized by health systems to ensure that patients receive comprehensive and synchronized management of their syndemic conditions.

## Figures and Tables

**Table 1:** Prevalence of DR-TB types among the study participants.

DR-TB Type	N (%)
RR	205 (46.1)
MDR	194 (43.6)
Pre-XDR	23 (5.2)
XDR	17 (3.8)
INH-resistant	6 (1.3)
Total	445 (100)

**Table 2:** Logistic regression model.

	Coeff	OR	*p*-Value
Intercept	1.2869	3.6217	<0.0001
BMI category overweight	−0.1481	0.8623	0.4126
BMI category obesity	1.1277	3.0884	0.0408
BMI category underweight	−0.2964	0.7435	0.0394
HIV status positive	−0.8078	0.4458	<0.0001

Coeff = coefficient; OR = Odds ratio.

## Data Availability

Data can be requested from the corresponding author.
